# The conformation change of Bcl-2 is involved in arsenic trioxide-induced apoptosis and inhibition of proliferation in SGC7901 human gastric cancer cells

**DOI:** 10.1186/1477-7819-8-31

**Published:** 2010-04-20

**Authors:** Yihu Zheng, Mengtao Zhou, Aifang Ye, Qiu Li, Yongheng Bai, Qiyu Zhang

**Affiliations:** 1 Department of Surgery, The First Affiliated Hospital of Wenzhou Medical College, Wenzhou 325000, China; 2 Key Laboratory of Surgery, The First Affiliated Hospital of Wenzhou Medical College, Wenzhou 325000, China; 3 Department of Laboratory, The First Affiliated Hospital of Wenzhou Medical College, Wenzhou 325000, China; 4 Department of Internal Medicine, The First Affiliated Hospital of Wenzhou Medical College, Wenzhou 325000, China

## Abstract

**Background:**

Arsenic trioxide has been established as a first-line agent for treating acute promyelocytic leukemia. Experimental data suggest that arsenic trioxide also can have a potential use as chemotherapeutic agent for other malignancies. The precise mechanisms of action of arsenic trioxide have though not been elucidated. As the role of Bcl-2 in arsenic trioxide-mediated cell apoptosis and conformation change of Bcl-2 in response to arsenic trioxide treatment has not been studied. The aim of the present study was to determine whether conformation change of Bcl-2 is involved in the action of arsenic trioxide.

**Methods:**

Human gastric cancer SGC7901 cells were exposed to different concentrations of arsenic trioxide. Proliferation was measured by using the Kit-8 cell counting assay. Analysis of nuclear morphology was observed by DAPI staining. The apoptosis rates of cells treated with arsenic trioxide were analyzed by flow cytometry using Annexin V-FITC staining. The conformation change of Bcl-2 and Bax activation were detected by immunostaining and Western blot analysis. Total expression of Bcl-2 and Bax were examined by Western blot analysis.

**Results:**

Arsenic trioxide inhibited the growth of human gastric cancer SGC7901 cells and induced apoptosis. There were two Bcl-2 phenotypes coexisting in SGC7901 cells and the Bcl-2 cytoprotective phenotype could change into a cytodestructive phenotype following conformational change of Bcl-2, triggered by arsenic trioxide exposure. Bax activation might also be involved in arsenic trioxide-induced Bcl-2 conformational change. Arsenic trioxide did not change levels of total Bcl-2 expression, but up-regulated total Bax expression for the treatment time ranging from 3 to 24 hours.

**Conclusion:**

Arsenic trioxide induces apoptosis through induction of Bcl-2 conformational change, Bax activation and up-regulation of total Bax expression rather than affecting total Bcl-2 expression in human gastric cancer SGC7901 cells. The conformational change of Bcl-2 may be a novel described mechanism of arsenic trioxide-induced apoptosis in cancer cells.

## Background

Arsenic trioxide, one member of the three inorganic forms of arsenic, is formed by heating realgar, which is formed as an arsenic complex with sulfur. Although arsenic trioxide is highly toxic, it has been shown to have a therapeutic potential. It has for long been used as a drug in traditional Chinese medicine to treat a variety of diseases, including malaria, psoriasis, syphilis, rheumatosis and cancer [1-3]. Contemporary studies show that arsenic trioxide is an effective therapeutic agent for the treatment of various hematological malignancies and especially acute promyelocytic leukemia [4-7]. More recent experimental data have demonstrated that arsenic trioxide may have effects in the treatment of several other malignancies in the experimental setting, including gastric cancer, lung cancer, breast cancer, hepatocellular carcinoma, gallbladder carcinoma, and neuroblastoma [8-13]. However, arsenic trioxide exerts its effect through different cellular and physiological pathways. The mechanisms of action of arsenic trioxide related to the induction of apoptosis in cancer cells remain controversial. Arsenic trioxide affects the activities of Akt, JNK kinases, NF-κB, glutathione, calcium signaling, ROS, Caspases, as well as pro- and anti-apoptotic proteins [14-17]. Down-regulation of Bcl-2, an "anti-apoptotic" protein, has also been considered as one of its significant mechanism of action [12,18-20].

Bcl-2 is considered as an important anti-apoptotic member of the Bcl-2 family, its expression manifests either cytoprotective or cytodestructive phenotypes, depending on the cellular context [21]. The anti-apoptotic Bcl-2 family members Bcl-2 and Bcl-XL have hydrophobic properties on their surfaces, essential for their anti-apoptotic effect, whereas their BH3 domains are buried. In contrast, pro-apoptotic Bcl-2 family members have an exposed BH3 domain, which binds to the hydrophobic pockets of anti-apoptotic Bcl-2 members to inhibit their survival effect [22]. Subsequent research showed that the dual phenotypes of Bcl-2 are controlled by its protein conformation [23]. When the loop of Bcl-2 interacts with an external factor, the hydrophobic binding groove of Bcl-2 undergoes a large-scale realignment, resulting in exposure of its BH3 domain [23,24]. This conformational change is responsible for the conversion of Bcl-2 from a cytoprotective to a cytodestructive molecule.

The present study aimed at determining whether arsenic trioxide inhibits proliferation and induces apoptosis in SGC7901 human gastric cancer cells, accompanied by conformational changes of Bcl-2 and changes in total Bcl-2 levels.

## Methods

### Materials

Arsenic trioxide was purchased from Sigma. A 5 mM stock solution of arsenic trioxide was obtained by dissolving arsenic trioxide in 1.65 M NaOH and by diluting in PBS, followed by adjustment of the pH to 7.0. RPMI Medium1640 and FBS were purchased from Invitrogen. Ant-Bcl-2 antibody (sc-492), anti-Bcl-2 antibody (sc-7382), anti-Bax antibody (sc-7480), ant-Bax (6A7) antibody (sc-23959) and anti-β-actin antibody (sc-47778) were from Santa Cruz Biotechnology. Anti-Bcl-2 BH3 (AP1303a) was from Abgent. Goat anti-mouse and rabbit secondary antibody conjugated to horseradish peroxidase (A0216, A0208), Cy3-labeled anti-rabbit IgG (A0516), FITC-labeled anti-mouse IgG (A0568) and DAPI were purchased from Beyotime Institute of Biotechnology.

### Cell culture and treatment

SGC7901 human gastric cancer cells were purchased from Shanghai Institutes for Biological Sciences and cultured in RPMI Medium1640 containing 10% FBS in a humidified atmosphere containing 5% CO_2 _at 37°C. Cells were split every 2-3 days by trypsinization and centrifugation, followed by aspiration of the culture medium. Before arsenic trioxide exposure, cell density was adjusted to 1.5 × 10^4 ^cells per square centimeter.

### Proliferation Analysis

Proliferation was measured by using the Cell Counting Assay Kit-8 (Dojindo Molecular Technologies) according to the manufacturer's protocol. One hundred microliters of SGC7901 human gastric cancer cells were plated on 96-well plates at a density of 1.5 × 10^4 ^cells per square centimeter and cultured for 24 hours. Cells were starved for 24 hours by replacing the media with serum-free media containing 0.1% BSA, followed by exposure to different concentrations of arsenic trioxide (0 μmol/L, 5 μmol/L, 10 μmol/L, 15 μmol/L and 20 μmol/L) for 24 and 48 hours. Ten microliters of Cell Counting Assay Kit-8 solution was added to each well, the cells were incubated for another 2 hours, and the absorbance at 450 nm was measured by using a microplate reader (BioTek Instruments). The amount of the formazan dye, generated by the activities of dehydrogenases in cells, is directly proportional to the number of living cells. Inhibitory rate of cellular growth was calculated as the following formula: Inhibitory rate (%) = (1-A value in experimental group/A value in control group) × 100%. The 0 μmol/L group was used as black control group. The IC50 value (the concentration of the drug which is capable of bringing about 50% inhibition of cell survival) of the drug used for treatment was determined by plotting a graph with inhibitory rate of cell growth (Y-axis) against the concentrations of the arsenic trioxide (X-axis).

### Analysis of nuclear morphology by DAPI staining

Cells grew in 6 well plates at a seeding density of 1.5 × 10^4 ^cells per square centimeter and were then treated with 10 μmol/L arsenic trioxide in complete media for 24 hours. Cells were fixed with 4% paraformaldehyde prior to washing with PBS. Washed cells were then stained with 1 μg/ml DAPI for 15 min in the dark. Slides were viewed with a fluorescent microscope at 340-380 nm and ×1000 magnification (Carl Zeiss). Cells were evaluated as normal or apoptotic depending on morphological characteristics. Normal nuclei (smooth nuclear) and apoptotic nuclei (condensed or fragmented chromatin) were easily distinguished. Thus, analysis of nuclear morphology was observed in three independent experiments.

### Apoptosis Analysis

Cells treated with different concentrations of arsenic trioxide (0 μmol/L, 5 μmol/L, 10 μmol/L, 15 μmol/L, 20 μmol/L, 25 μmol/L and 30 μmol/L) in serum-free medium for 24 hours were collected and stained with Annexin V/propidium iodide (PI) using Vybrant apoptosis assay kit No. 2 (Molecular Probes) and analyzed by flow cytometry. The 0 μmol/L group was used as black control group.

### Immunofluorescence microscopy

Cells treated with 15 μmol/L arsenic trioxide for 12 hours were cultured in serum-free medium overnight on glass coverslips. The cells were fixed in 4% paraformaldehyde in PBS for 15 min and washed twice with PBS. The cells were then permeabilized with 1% Triton X-100 in PBS for 5 min. Fixed cells were preincubated for 45 min in PBS containing 5% bovine serum albumin at room temperature, followed by incubation with various primary antibodies at 4°C overnight and detected by Cy3-labeled anti-rabbit IgG (1:300) or FITC-labeled anti-mouse IgG (1:300) at room temperature for 2 hours. Cells were stained with 1 μg/ml DAPI to visualize the nuclei. The images were taken under a fluorescent microscope. The primary antibodies included ant-Bcl-2 antibody (sc-492) (Santa Cruz, 1:200), anti-Bcl-2 BH3 antibody (Abgent, 1:200) and anti-Bax(6A7) antibody (sc-23959) (Santa Cruz, 1:200).

### Protein extraction and western blot analysis

Cells were treated with 15 μmol/L arsenic trioxide for different time. Both adherent and floating cells were harvested and lysed with Mammalian Protein Extraction Reagent (Pierce) according to the manufacturer's protocol. Equal amounts of protein were separated by SDS-PAGE or Native-PAGE, and then transferred onto a PVDF membrane (Millipore). The membrane was blocked for 1 hour in a non-fat dried milk solution containing 1% Tween-20. The membrane was then incubated with various primary antibodies overnight at 4°C, followed by incubation with anti-mouse (1:5.000) secondary antibodies for 1 hour. Finally, protein bands were detected by using the Chemiluminescent Substrate (HRP) Kit from Pierce. The dilutions of the primary antibodies were anti-Bcl-2 antibody (sc-7382) in 1:800, anti-Bax antibody (sc-7480) in 1:800, anti-Bcl-2 BH3 antibody in 1:500, anti-Bax (6A7) antibody in 1:500. The blots were reprobed with anti-β-actin antibody for loading control.

### Statistical analysis

The results of each series of experiments (performed in triplicates) were expressed as the mean values ± standard deviation of the mean (SD). Statistical significance of differences between groups was analyzed by using ANOVA analysis. *P *< 0.05 was considered statistically significant.

## Results

### Proliferation Analysis

**Figure 1:**
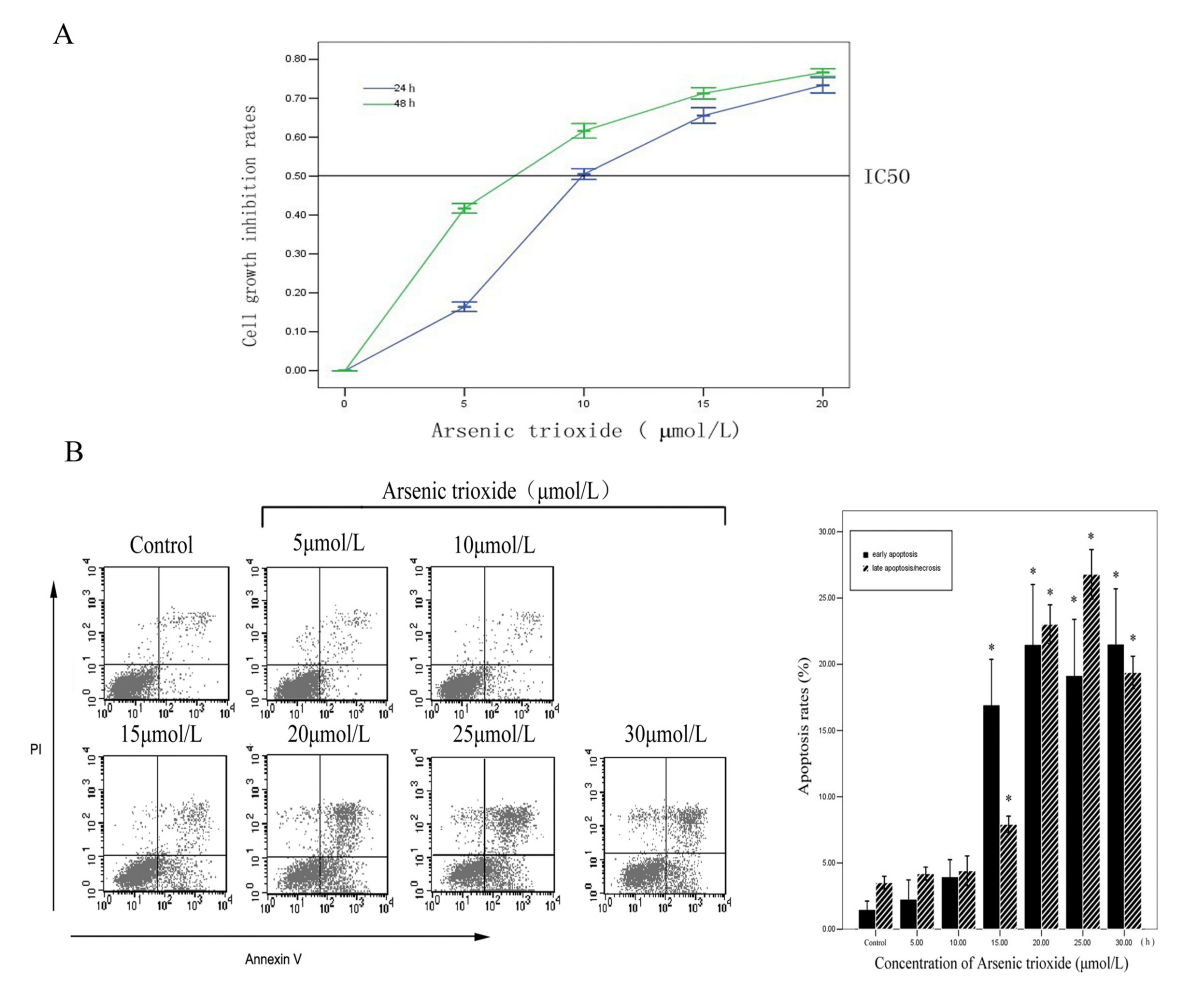
**A. Inhibitory effects of arsenic trioxide on SGC7901 cell**. **B **The effects of arsenic trioxide on early and late apoptosis/necrosis of SGC7901 cell. (* represents *p *< 0.05 compared to the black control group and arsenic trioxide treated groups).

SGC7901 cells were treated with different concentrations of arsenic trioxide (5 μmol/L, 10 μmol/L, 15 μmol/L and 20 μmol/L) at 24 and 48 hours. The inhibitory rates of cell growth were 16.50 ± 0.55%, 50.83 ± 0.75%, 65.50 ± 1.05%, 73.50 ± 1.05%; 41.83 ± 0.75%, 61.67 ± 0.82%, 71.17 ± 0.75%, 76.67 ± 0.82%, respectively. By using curve fitting, the IC50 was about 10 μmol/L for 24 hours. Arsenic trioxide obviously could inhibit the proliferation of SGC7901 cells in concentration and time-dependent manner (Fig. 1A).

### Morphologic characteristic of apoptosis

**Figure 2:**
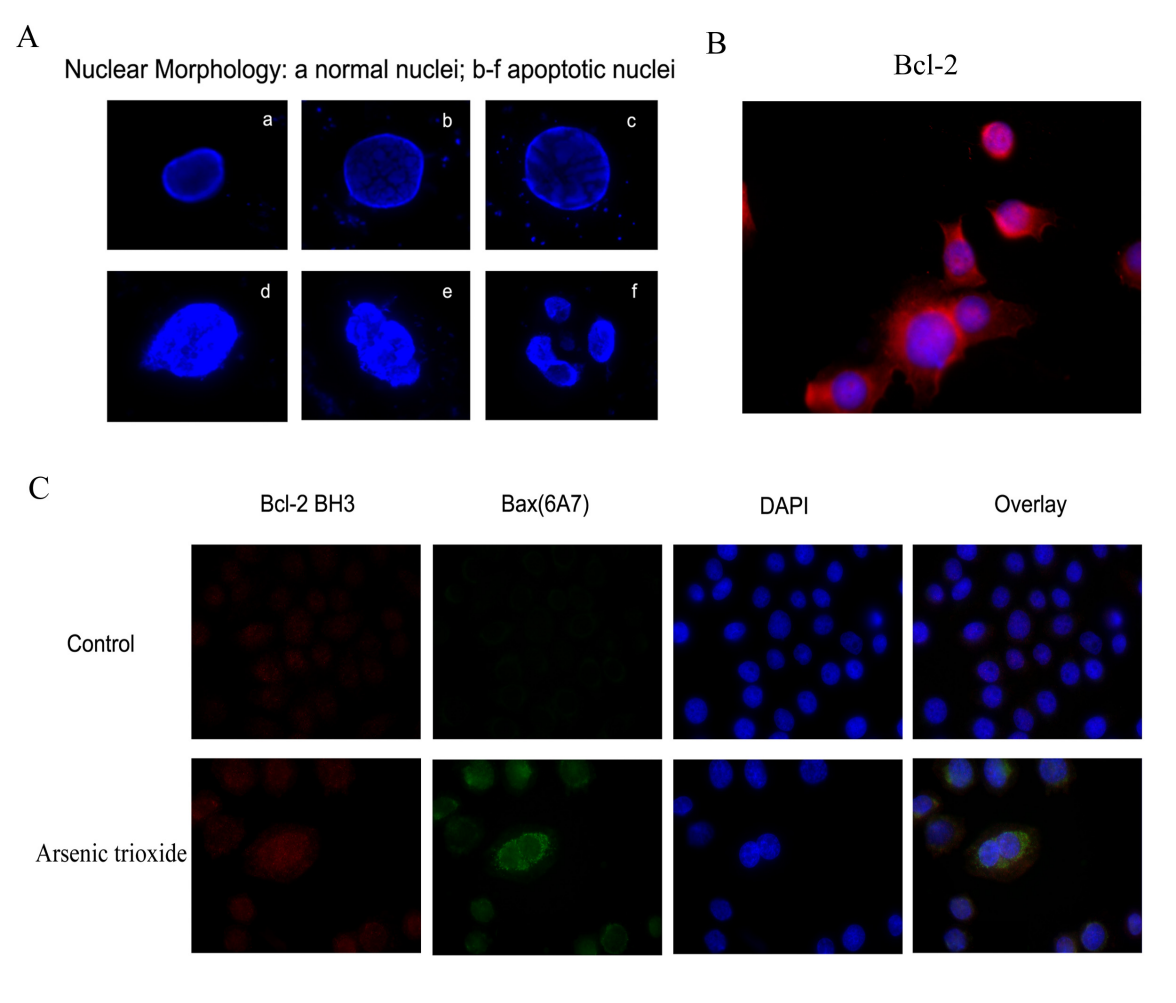
**A. Nuclear morphologic changes showing features of apoptosis in SGC7901 cells after treatment with arsenic trioxide for 24 hours**. (a) Untreated SGC7901 cells; (b-f) 15 μmol/L arsenic trioxide-treated SGC7901 cells. The cells were stained using DAPI staining. **B**. Untreated SGC7901 cells stained by anti-Bcl-2 N terminus antibody. **C **SGC7901 cell stained by anti-Bcl-2 BH3 and anti-Bax(6A7) antibody before and after exposure to 15 μmol/L arsenic trioxide for 12 hours.

Nuclear morphology analysis showed characteristic apoptotic changes, such as convoluted nuclei with cavitations, clumps of chromatin abutting to inner regions of the nuclear envelope between the nuclear pores, breakdown of nuclear envelope, chromatin condensation and dissociation of DNA fragments in SGC7901 cells after treatment with arsenic trioxide for 24 hours (Fig. 2A).

### Apoptosis Analysis

SGC7901 cells were treated with different concentrations of arsenic trioxide (0 μmol/L, 5 μmol/L, 10 μmol/L, 15 μmol/L, 20 μmol/L, 25 μmol/L and 30 μmol/L) for 24 hours. The early and late apoptosis/necrosis rates were 11.49 ± 0.63%, 2.28 ± 1.46%, 3.97 ± 1.28%, 16.94 ± 3.42%, 21.50 ± 4.51%, 19.16 ± 4.21%, 21.53 ± 4.16%; 3.52 ± 0.49%, 4.21 ± 0.48%, 4.42 ± 1.12%, 7.92 ± 0.61%, 23.02 ± 1.46%, 26.80 ± 1.86%, 19.39 ± 1.23%, respectively, which suggested that arsenic trioxide induce apoptosis (Fig. 1B).

### Arsenic trioxide induced Bcl-2 conformational change and Bax activation

**Figure 3:**
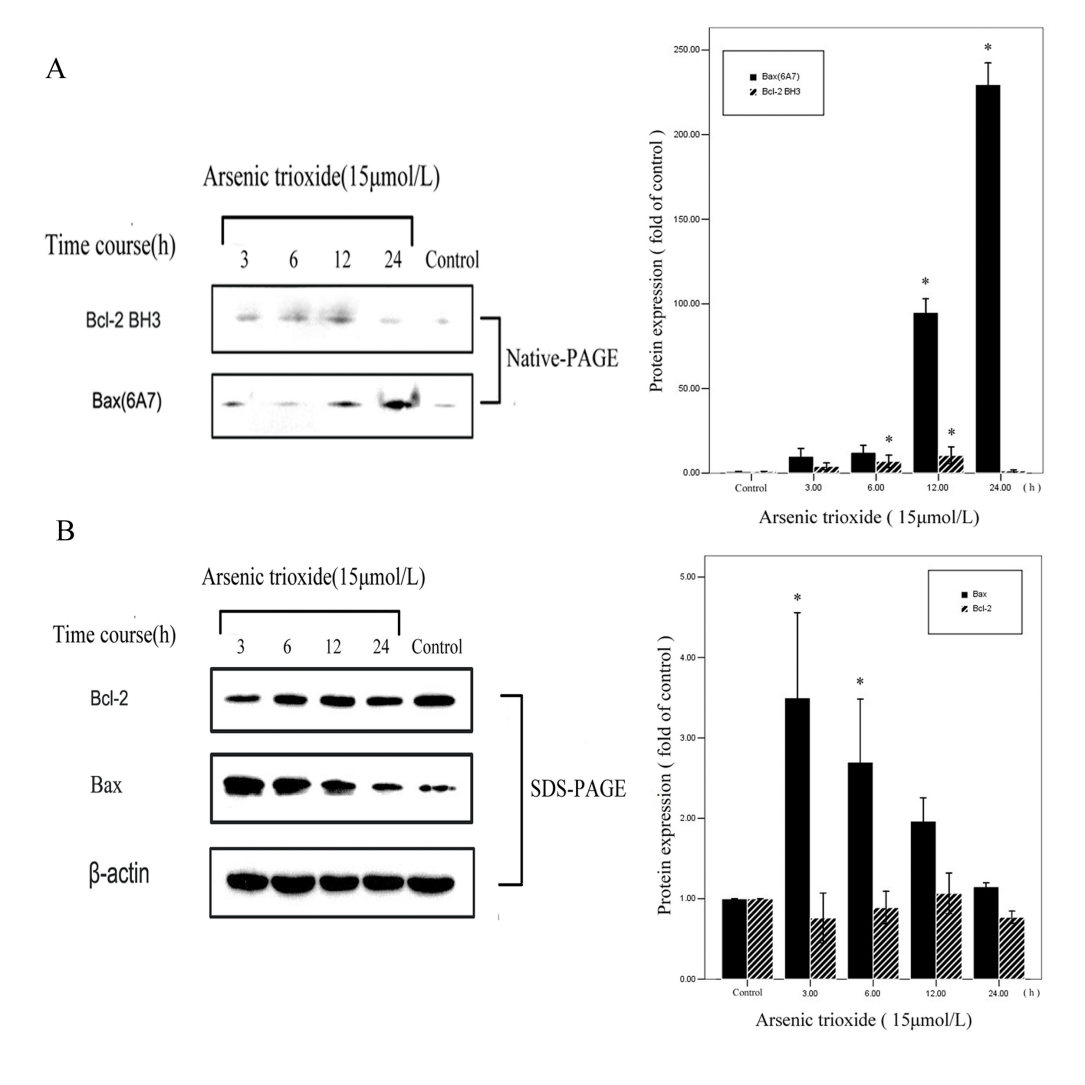
**A. The expression of BH3 exposed Bcl-2 and activated Bax after exposed to 15 μmol/L arsenic trioxide in SGC7901 cell**. **B **The expression of total Bcl-2 and total Bax after exposure to 15 μmol/L arsenic trioxide in SGC7901 cell. (* represents *p *< 0.05 between black control group and arsenic trioxide treated groups).

SGC7901 cells were strongly stained by anti-Bcl-2 N terminus antibody. It showed that SGC7901 cells highly expressed total Bcl-2 protein (Fig. 2B). Moreover, an enhanced immunostaining by anti-Bcl-2 BH3 antibody, as compared to the "black control group" (0 hour), was observed in SGC7901 cells treated with 15 μmol/L arsenic trioxide for 12 hours using immunofluorescence. The black control group (0 hour) did not immunostain by the anti-Bax (6A7) antibody, suggesting that Bax was inactive in the cells. However, SGC7901 cells treated with 15 μmol/L arsenic trioxide displayed strong immunostaining with the anti-Bax (6A7) antibody, demonstrating that arsenic trioxide could activate Bax (Fig. 2C). After treatment of 15 μmol/L arsenic trioxide for the indicated times (0 hour, 3 hours, 6 hours, 12 hours and 24 hours), Western blot showed that the expression of BH3 domain exposed Bcl-2 had an upward trend and reached a peak at 12 hours and the difference as compared with 0 hour was statistically significant (*P *< 0.05). By time, the activated Bax also presented an upward trend and reached a peak at 24 hours and the difference compared to 0 hour was statistically significant (*P *< 0.05) (Fig. 3A). Arsenic trioxide-treated SGC7901 cells, detected by western blot and stained by both anti-Bcl-2 (BH3) and anti-Bax (6A7) antibodies, express conformational change of Bcl-2, which may play a role in arsenic trioxide-induced apoptosis and Bax activation.

### Arsenic trioxide did not affect total Bcl-2 expression, but up-regulated total Bax expression

After 15 μmol/L arsenic trioxide exposure for various times (0 hour, 3 hours, 6 hours, 12 hours and 24 hours), the change in total Bcl-2 expression was unconspiciuous and the differences compared to the different groups did not statistically differ (*P *> 0.05). Total Bax had a higher expression and reached a peak at 3 hours and the differences compared to 0 hour was statistically significant (*P *< 0.05), though the levels descended at 3 hours (Fig. 3B). The results showed that arsenic trioxide did not cause any apparent change in levels of Bcl-2, but Bax expression was up-regulated for treatment times ranging from 3 to 24 hours.

## Discussion

Arsenic is a well-known environmental toxic and carcinogenic substance, and an effective chemotherapeutic drug. Due to the dual capability of arsenic, the agent carries significant risks for medical applications. The underlying mechanisms are, however, not fully understood. Arsenic exerts its effect by inhibiting the activities of several enzymes, especially those involved in cellular signaling pathways and DNA synthesis and repair. During the past centuries, a number of arsenic compounds have been used as medicines. Arsenic trioxide, one form of arsenicals, has been used in a variety of ways over the past hundred years, but most commonly in the treatment of malignancies. Owing to the impressive effects of arsenic trioxide in hematological cancers and solid tumor cells in vitro, the mechanisms of arsenic trioxide-mediated cell death have recently come under increasing scrutiny. Arsenic trioxide may be a promising candidate for the treatment of other malignancies. The combination therapy of arsenic trioxide and other chemotherapeutic agents have been applied experimentally for treatment of refractory malignant tumors.

In the current study, we observed that arsenic trioxide had a strong anti-proliferative effect, most likely by induction of apoptosis, on human gastric cancer SGC7901 cells in a dose and time dependent manner. As has previously been reported, the cellular and biochemical effects of arsenic were performed using concentrations greater than 5 μmol/L, often 50 μmol/L, and the 50% inhibitory concentration (IC50) of arsenic trioxide on proliferation of SGC7901 cells was about 10 μmol/L for 24 hours. Maybe it was much too high than relevant to therapeutic levels (1 to 2 μmol/L) [25,26]. However, from the 24 and 48 hours curve fitting, we could suppose that the 50% inhibitory concentration (IC50) for 72 hours may be similar to clinically therapeutic levels, which also has been described by others [8,9,27]. This suggests that SGC7901 human gastric cancer cells are sensitive to arsenic trioxide.

The mechanisms of arsenic trioxide-induced anti-proliferation have been extensively investigated. Apoptosis appears to be the main phenomenon resulting in significant cell death and cell growth inhibition. Arsenic trioxide is known to modulate multiple signal transduction pathways, including inhibition of telomerase activity, induction of reactive oxygen species release, and inhibition of survival pathways involving extracellular signal regulated kinase, Akt, calcium signaling and NF-κB activities [14-17,28]. Interestingly, the apoptotic effect of arsenic trioxide largely depends on a Bcl-2-controlled pathway [12,18-20].

Bcl-2, an anti-apoptotic Bcl-2 family member, for which an increased expression has been associated with a more aggressive malignant phenotype and drug resistance to various categories of chemotherapeutic drugs in malignancies. Small molecule inhibitors of the Bcl-2 family proteins, designed to bind the hydrophobic groove of anti-apoptotic Bcl-2 proteins in place of BH3-only proteins, are potential agents to treat cancers. They can oligomerize Bax or Bak, which subsequently depolarize in the mitochondrial membrane potential to release cytochrome c and induce apoptosis [29]. Agents targeting anti-apoptotic Bcl-2 family members have preclinical activity as single agents and also affect combination with other anti-neoplastic agents. Recent researches have demonstrated that Bcl-2 could manifest opposing phenotypes, induced by interactions with proteins, such as Nur77, suggesting novel strategies for regulating apoptosis in cancers and other diseases [30]. This phenotype change of Bcl-2 is controlled by its protein conformational change. When the loop of Bcl-2 interacts with an external factor, the hydrophobic binding groove of Bcl-2 undergoes a large-scale realignment, resulting in exposure of its BH3 domain [21,22]. It was also reported that paclitaxel could directly target Bcl-2 in the loop domain, mimics activity of Nur77, thereby facilitating the initiation of apoptosis [31].

Whether Bcl-2 phenotype changes phenomenon occur in arsenic trioxide-induced cell apoptosis is still unknown. In the present study, we used anti-Bcl-2 BH3 antibody to detect the conformational change of Bcl-2. When Bcl-2 undergoes conformational change, the hydrophobic binding groove of Bcl-2 gives rise to a large-scale realignment, resulting in exposure of its cryptic BH3 domain and can be recognized by Bcl-2 BH3 antibody. We used Bax (6A7) antibody to detect the activated Bax, Bax undergoes a conformational change and oligomerization during early apoptosis, which can be followed by exposure of cryptic antibody epitopes (the N-terminal residues 1-21). This type of Bax can be recognized by anti-Bax (6A7) antibody. The novel finding from this work was that SGC7901 cells highly expressed Bcl-2, but they were weakly stained by the anti-Bcl-2 BH3 antibody, suggesting that there were two Bcl-2 phenotypes coexisting in SGC7901 cells and mostly Bcl-2 was anti-apoptotic. The results showed that Bcl-2 anti-apoptotic phenotype could change into a pro-apoptotic phenotype following exposure to arsenic trioxide. Also Bax activation was involved in arsenic trioxide-induced conformational change of Bcl-2 by immunostaining SGC7901 cells with anti-Bax (6A7) antibody that recognizes activated Bax. Arsenic trioxide caused no apparent change in the levels of Bcl-2, but up-regulated Bax for treatment times ranging from 3 to 24 hours. Thus, Bcl-2 conformational change, Bax activation and up-regulation of total Bax expression involved arsenic trioxide-induced apoptosis rather than affecting total Bcl-2 expression in human gastric cancer SGC7901 cells.

Although the anti-apoptotic effect of Bcl-2 is well established, the role of Bcl-2 in cancer response to therapy and drug resistance has not been completely explored. The mechanism how it promotes cell death has recently gained increasing interest. In general, over-expression and up-regulation of Bcl-2 has been associated with resistance to chemotherapy in various human cancers [29,32], and many studies have shown that over-expression of Bcl-2 is a poor prognostic factor in various cancers. It was found that Bcl-2 expression tended to be associated with a worsened survival in olfactory neuroblastoma (ONB) [33]. Also the expression of Bcl-2 and Bax proteins, evaluated by immunohistochemical staining in specimens from 110 patients with oral squamous cell carcinoma (OSCC) showed that the 5-year survival rate was significantly higher in patients with a ratio of Bcl-2/Bax ≤ 1 as compared to those with Bcl-2/Bax > 1 [34]. On the opposite side, high Bcl-2 expression also correlated with favorable parameters and a better prognosis in other cancers. A recent systematic review of the literature showed that over-expression of Bcl-2 was a good prognostic factor for survival in patients with non-small cell lung cancer [35]. Bcl-2 expression also correlated with a favorable prognosis in colorectal cancer [36], and with improved overall survival rate in oral squamous cell carcinoma [37].

Our finding of conformational change of Bcl-2 in SGC7901 cells following exposure to arsenic trioxide is important for founding an explanation accounting for the opposing biological activities of Bcl-2. This may also represent that arsenic trioxide may be a promising candidate for the future treatment of malignancies that over-express endogenous Bcl-2, though substantial experimental and clinical research remains to validate its potential value.

## Conclusion

Our results show that arsenic trioxide is an effective anti-cancer agent with potential for human gastric cancer. Arsenic trioxide can reduce proliferation and induce apoptosis in SGC7901 human gastric cancer cells. There are two Bcl-2 phenotypes coexisting in SGC7901 cells and the Bcl-2 cytoprotective phenotype can change into a cytodestructive phenotype following arsenic trioxide exposure. Also Bax activation is involved in arsenic trioxide-induced conformational change of Bcl-2 in SGC7901 cells. The conformational change of Bcl-2 may be the new mechanism explaining arsenic trioxide-induced apoptosis, other than the ones affecting the total Bcl-2 expression in some cancer cells.

## Abbreviations

As_2_O_3_: arsenic trioxide; APL: acute promyelocytic leukemia; JNK: c-jun terminal kinase; NF-κB: nuclear factor κB; ROS: reactive oxygen species; PBS: phosphate buffered saline; FBS: fetal bovine serum; HSP: heat shock proteins; PVDF: polyvinylidene fluoride; SDS-PAGE: sodium dodecyl sulfate-polyacrylamide gel electrophoresis.

## Competing interests

The authors declare that they have no competing interests.

## Authors' contributions

YZ and AY are researchers working in cancer biology and carried the study. QL and YB undertook the Statistical analysis. QZ along with MZ designed the work and interpreted the results. QZ and MZ contributed to the writing of the manuscript. All the authors read and approved the final manuscript.
